# Atractylenolide I modulates ovarian cancer cell-mediated immunosuppression by blocking MD-2/TLR4 complex-mediated MyD88/NF-κB signaling in vitro

**DOI:** 10.1186/s12967-016-0845-5

**Published:** 2016-04-27

**Authors:** Hong Liu, Guonan Zhang, Jianming Huang, Shiqi Ma, Kun Mi, Jia Cheng, Yi Zhu, Xiao Zha, Wei Huang

**Affiliations:** Department of Obstetrics and Gynecology, West China Second University Hospital of Sichuan University, No. 20, Section 3 of South People’s Road, Chengdu, 610041 China; Department of Gynecologic Oncology, Sichuan Cancer Hospital, No.55, Section 4 of South People’s Road, Chengdu, 610041 China; Department of Biochemistry and Molecular Biology, Sichuan Cancer Institute, No.55, Section 4 of South People’s Road, Chengdu, 610041 China; Department of Ultrasound, Sichuan Cancer Hospital, No.55, Section 4 of South People’s Road, Chengdu, 610041 China

**Keywords:** Epithelial ovarian cancer, TLR4/MD-2 complex, MyD88/NF-κB signaling, Atractylenolide I, Immunosuppressive cytokines, Indoleamine 2,3-dioxygenase

## Abstract

**Background:**

TLR4/MD-2 complex-mediated MyD88-dependent activation of NF-κB and Akt promotes tumor-associated immunosuppression in epithelial ovarian cancer (EOC) via induction of immunesuppressive cytokines and indoleamine 2,3-dioxygenase (IDO). Atractylenolide I (AO-1) is a naturally occurring sesquiterpene lactone known to change the conformational ensemble of human MD-2 on EOC cells. This study examined the modulation by AO-1 of TLR4/MD-2 complex-mediated MyD88/NF-κB signaling.

**Methods:**

The expression and activation of NF-κB, Akt and IDO1 by MyD88^+^ EOC SKOV3 cells was determined using western blot; the TLR4/MD-2 complex on SKOV3 cells and the phenotype of T lymphocytes were determined using flow cytometry; IDO activity was evaluated by measuring l-kynurenine; Immunesuppressive cytokines were detected using ELISA; T‐cell proliferation to mitogen stimulation was assessed by MTT assay; the cytotoxicity of lymphocytes and NK cells was measured using LDH-cytotoxicity assay.

**Results:**

AO-1 could down-regulate expression of TLR4/MD-2 complex, resulting in downregulation of MyD88/NF-κB signaling and activation of NF-κB, Akt and IDO1 and secretion of IL-6, TGF-β1, VEGF and IL-17A by EOC SKOV3 cells, and further reduce increased levels of regulatory T cells (Treg cells) and improve decreased proliferative response and antitumor cytotoxicity of T lymphocytes exposed to EOC SKOV3 cell supernatant.

**Conclusion:**

AO-1 may reverse EOC cell-mediated immunosuppression through blocking TLR4/MD-2 complex-mediated MyD88/NF-κB signaling.

## Background

Epithelial ovarian cancer (EOC) is the fifth leading cause of death among women with gynecologic malignancies [1]. Recent research has suggested that EOC is capable of escaping the immune system due to interaction between cancer cell and host immune cell in tumor microenvironment. EOC cells constitute an immunosuppressive environment that promotes tumor growth, progression, and immune evasion [2]. Cancer-induced immunosuppression is an intractable problem as it impairs the response to immunotherapy [3]. Increasing evidence suggests that cancer cells have usurp acquired many properties characteristic of immune cells, allowing them to communicate and more importantly, modulate the immune responses and escape immune elimination for its own survival and growth [4]. The intracellular signaling components of Toll-like receptors (TLRs) constitute an important cellular signal pathway which induces tumor immnuosuppression and chemoresistance. The activation of TLRs signaling in tumor cells induces the production of various immunosuppressive cytokines to impair dendritic cells (DCs) ability to stimulate antitumor T cells and to induce various immunosuppressive cells, then resulting in immune incompetence in the tumor microenvironment [5, 6]. Therefore, it is important to elucidate molecular mechanism of the immunosuppression by EOC, and to develop strategies to restore immunocompetence in EOC patients.

It has been shown that EOC cells bear TLR4 and MyD88, an adapter coupling TLR4 and that TLR4/MyD88 signaling induces the synthesis of immunosuppressive cytokines and facilitates tumor progression and immune evasion [7]. MyD88 is expressed in approximately 70 % of patients with EOC and has been identified as an independent factor of poor prognosis [8, 9]. Signaling through TLR4 requires concomitant expression of an accessory protein, myeloid differentiation protein-2 (MD-2), which is functionally an essential component of the TLR4 signaling complex with an indispensable role for the initiation of TLR4/MyD88 signaling [10–12]. TLR4/MD-2 complex is required for activation of MyD88-dependent NF-κB pathway [13–15] in MyD88 expressing EOC cells. NF-κB is one of the main intracellular pathways mediating the induction of immunosuppressive cytokine and enzyme expression following TLR4 activation [16]. It has been found that EOC cells constitutively express interleukin (IL)-6, IL-4, IL-10, IL-17A, VEGF, TGF-β1 and indoleamine 2,3-dioxygenase [17–29]. These immunosuppresants produced by EOC cells represents a significant mechanism of tumor immune escape and tolerance through increasing Treg cells and suppressing NK cell function, T cell activation and proliferation [2, 30]. Thus, the effective immunotherapy for EOC may be achieved by reversing EOC cell-mediated immunosuppression.

Atractylenolide I (AO-1) is a natural sesquiterpene lactone extracted from Atractylodes macrocephala Koidz [Family: Compositae] which possesses immune regulation in Chinese traditional medicine [31], and is known to antagonize TLR4-mediated production of proinflammatory cytokines including NO, TNF-α, IL-6, IL-1β, and VEGF in some immune cells [32]. Previously we showed that AO-1 can significantly reduce expression of TLR4 and MD-2 by EOC cells [33]. These data suggest that AO-1 may make the molecular conformation of MD-2 changed and interrupt the assembly of TLR4/MD-2 complex, and then block MyD88-dependent signaling pathway. However, it is unknown whether MyD88 act autonomously or require a TLR4/MD-2 complex to engage these downstream pathways. Thus, research into immunotherapy targeting TLR4/MD-2 complex-mediated MyD88/NF-κB and Akt pathways of EOC cells will probably become focused on an approach to combination immunotherapy that simultaneously intensify anti-tumor immune responses while reversing tumor immune suppression. In this study, we evaluated the role of TLR4/MD-2 complex in the immunosuppression caused by human EOC, and the immune modulation of AO-1 targeting TLR4/MD-2 complex-mediated MyD88-dependent activation of NF-κB through blocking MD-2 binding to TLR4. Our primary findings showed that AO-1 could inhibit TLR4/MD-2 complex-mediated MyD88-dependent activation of NF-κB and Akt, and reduce the expression of immunosuppressive molecules by SKOV3 cells and the level of Treg cells induced by the supernatant of SKOV3 cells, and enhance proliferative response and cytotoxicity of T lymphocytes. These findings suggest that AO-1 could modulate EOC cell-mediated immunosuppression by targeting TLR4/MD-2 complex on EOC cells and that AO-1 is a potential inhibitor of TLR4/MD-2 complex. Therefore, TLR4/MD-2 complex is an attractive target for the development of effective EOC immunotherapeutic strategies for EOC patients whose tumors express TLR4/MD-2/MyD88 and NF-κB signaling pathways.

## Methods

### Compounds and reagents

Atractylenolide I, (4aS,8aS)-3,8a-dimethyl-5-methylidene-4a,6,7,8-tetrahydro-4H-benzo[f][1] benzofuran-2-one (CAS No.73069-13-3, MF C_15_H_18_O_2_, m.w. (g/mol) 230.3022, HPLC 98 %) was purchased from Chengdu Best-Reagent Co. Ltd. (Chengdu, China); lipopolysaccharide (LPS), phytohemagglutinin (PHA), MTT (3-[4,5-dimethylthiazol-2-yl]- 2,5-diphenyl tetrazolium bromide), l-kynurenine and 4-dimethylaminobenzaldehyde were purchased from Sigma Chemical Co. (St. Louis, MO, USA); the rabbit polyclonal antibodies to NF-κB p65, phospho-NF-κB p65 (S536)(ab76302), Akt and phospho-Akt (S129) (ab133458), anti-GAPDH antibody [EPR6256] (ab128915) and anti-TLR4/MD-2 complex antibody[7E3](FITC)(ab105855) were purchased from Abcam plc.(Cambridge, MA, USA); Peroxidase-Conjugated AffiniPure Goat Anti-Rabbit IgG was purchased from ZSGB-BIO, Inc. (Beijing,China); ELISA kits for IL-6, IL-4, IL-10, IL-17A, TGF-β1 and VEGF were purchased from R&D Systems, Inc. (Minneapolis, MN 55413, USA); CD4-FITC/CD8-PE/CD3-PerCP (Catalog Number: 340298) for determining CD4/CD8/CD3 counts of T lymphocytes and FoxP3 Staining Kit—FoxP3-PE/CD4-FITC/CD25-APC (Catalog Number: 560133) for determining counts of Treg cells were purchased from Becton, Dickinson and Company (BD Biosciences, USA); CytoTox 96^®^ Non-Radioactive Cytotoxicity Assay (G1781) was purchased from Promega Corporation (Madison, WI, USA).

### Cell lines and cultures

Human EOC SKOV3 cell line (MD-2^+^/TLR4^+^/MyD88^+^, derived from the ascites of a patient with advanced, metastatic EOC and resistant to most cytotoxic drugs) and human K562 cell line (lacking the MHC, derived from a female patient with chronic myeloid leukemia) and Raji cell line (MHC^+^, derived from B-lymphocyte of a male patient with Nigerian Burkett’s lymphoma) were purchased from the Committee on Type Culture Collection of Chinese Academy of Sciences (CTCCCAS, Shanghai, China). Cell lines were maintained in RPMI-1640 medium (GIBCO) supplemented 10 % heat inactivated fetal calf serum (FCS), 2 mmol/L l-glutamine, 100 U/mL penicillin and 40 IU/mL gentamycin (complete RPMI-1640 medium) at 37 °C in a humidified atmosphere of 5 % CO_2_ and 95 % air. Subconfluent cells (80 %) were passaged with a solution containing 0.25 % trypsin and 0.5 mmol/L EDTA. Cell lines were tested for mycoplasma and confirmed to be negative.

### Preparation of monocyte-free lymphocytes

The peripheral blood mononuclear cells (PBMCs) were prepared from heparinized venous blood of a healthy female adult donor by centrifugation over a Ficoll-Hypaque(1.077 g/mL) gradient. PBMCs were resuspended in complete RPMI-1640 medium, and incubated in 60-mm glass culture flasks at 37 °C in a 5 % CO_2_. After 4 h, the nonadherent cells devoid of contaminating monocytes, i.e., lymphocytes, were harvested to be used for this study.

### Preparation of SKOV3 cell supernatants

1 × 10^6^ SKOV3 cells were seeded into 6-well (Corning Costar) in a final volume of 2 mL and preincubated at 37 °C in a 5 % CO_2_ for 24 h, then replaced with RPMI-1640 medium containing 2 % FCS with addition of the specified concentration of AO-1, LPS and 1-MT and incubated at 37 °C in a 5 % CO_2_ for 48 h, and the culture supernatants were harvested by centrifugation at 1000*g* for 10 min at 4 °C min, and stored at −80 °C until use for analysis.

### Western blot

SKOV3 cells were lysed in RIPA buffer [1 % Triton X-100, 150 mmol/L NaCl, 1 mmol/L EGTA, 50 mmol/L Tris–HCl, 0.1 % sodium dodecyl sulfate (SDS), 1 % sodium desoxycholate and phenylmethylsuphonyl fluoride (PMSF)]. Proteins separated by SDS-PAGE were electrotransfered on polyvinylidene difluoride (PVDF) membranes. After blocking, the membrane was incubated with the primary antibody at 4 °C overnight, and washed three times and incubated with Peroxidase-Conjugated AffiniPure Goat Anti-Rabbit IgG secondary antibody (1:100,000 dilution) at 37 °C for 1 h, and then developed in an electrochemiluminescence (ECL) detection system (ImageQuant™LAS500,GE). GAPDH antibody as loading control was used to normalize the levels of protein detected.

### ELISA

Enzyme-linked immunosorbent assay (ELISA) was used to determine cytokine IL-6, IL-10, IL-4, IL-17A, TGF-β1 and VEGF according to the manufacturer’s protocols of ELISA kits. Briefly, 100 μL of the culture supernatant of SKOV3 cells, the standard or the control was added to each well in 96-well ELISA plate (R&D) and incubated for 2 h at room temperature. Each well was aspirated and washed three times with Wash Buffer, then 200 μL of the antibody specific for cytokine conjugated to horseradish peroxidase was added to each well, incubated for 2 h at room temperature, aspirated and washed three times with Wash Buffer. 200 μL of the substrate solution to each well, and incubated in the dark for 20 min at room temperature, and then 50 μL of the stop solution was added to each well. The absorbance was determined using Infinite M200 Pro TECAN-Reader at 450 nm, with the correction at wavelength set 570 nm.

### Measurement of l-kynurenine

IDO activity was evaluated by measuring the levels of tryptophan metabolite, l-kynurenine, present in the supernatant of SKOV3 cells with Ehrlich’s reagent (1.2 % 4-dimethylaminobenzaldehyde in glacial acetic acid). Briefly, 150 μL of the culture supernatants of SKOV3 cells were added to each well of a 96-well round-bottom culture plate, and after addition of 10 μL 30 % (v/v) trichloroacetic acid to each well, the plate was incubated for 30 min at 50 °C to hydrolyze N-formylkynurenine to kynurenine, and centrifuged at 1500*g* for 10 min; 100 μL of supernatant was transferred to the corresponding wells of a 96-well flat-bottom plate and mixed with 100 μL of freshly prepared Ehrlich’s reagent and incubated for 10 min at room temperature. Absorbance was read at 492 nm using Infinite M200 Pro TECAN-Reader, with a blank that containing culture media only and purified l-kynurenine (0–100 μmol/L) was used as a standard.

### Flow cytometry for TLR4/MD-2 complex expression by SKOV3 cells

TLR4/MD-2 complex expression by SKOV3 cells were determined using TLR4/MD-2 complex staining kit [7E3](FITC) according to the manufacturer’s protocols. Briefly, SKOV3 cells treated with LPS (1 μg/mL) and AO-1 (10, 50 and 100 μmol/L) for 6 h were harvested with 0.25 % trypsin digestion and washed three times with PBS containing 2 % FCS (FCS) by centrifugation at 500*g* for 5 min at 4 °C, and resuspended in 100 μL of antibody diluent, and incubated with FITC anti human TLR4/MD-2 complex mAb for 30 min at 4 °C, then centrifuged at 500*g* for 5 min at 4 °C and the supernatants were removed and fixed with 4 % paraformaldehyde in PBS (10 mmol/L, pH 7.4) for overnight at 4 °C, and washed twice with washing buffer by centrifugation at 500*g* for 5 min at 4 °C. The stained cells were resuspended in 1 mL of FACS buffer and were analyzed on FACSCanto II Flow Cytometer (Becton–Dickinson).

### Flow cytometry for the phenotype of T lymphocytes

The phenotype of T lymphocytes exposed to SKOV3 cell supernants for 24 h was assessed by flow cytometry (FCM). For CD3^+^/CD4^+^/CD8^+^ counts, 20 μL TriTEST CD4-FITC/CD8-PE/CD3-PerCP antibody was added to a test tube, and then 20 μL of lymphocyte suspension containing 1 × 10^6^ cells was added to the tube, and mixed gently. After incubation in the dark for 15 min at room temperature, 450 μL of 1 × BD FACS lysing solution was added to the tube. Gating for T-cells was performed using CD3^+^ cells. Cell Quest software (BD Bioscience) was used to determine the percentage of CD4^+^ and CD8^+^ cells in the CD3^+^ cells; For CD4^+^/CD25^+^/Foxp3^+^ Treg cells, 20 μL of FITC Mouse Anti-Human CD4 and 20 μL APC Mouse Anti-Human CD25 were added to a test tube, and then 100 μL of lymphocytes suspension containing 1 × 10^6^ cells was added to the tube, mixed fully and incubated in the dark for 30 min at 2–8 °C, washed once with 2 mL of pre-cooling PBS by centrifugation at 1000 rpm for 5 min at 4 °C. One milliliter of 1 × Fix/Perm buffer was added to the tube, and incubated in the dark for 40–50 min at 2–8 °C, then washed twice with 2 mL of 1 × Perm/Wash buffer. The cells were resuspended in 100 μL of 1 × Perm/Wash buffer, and 100 μL of PE Mouse Anti-Human Foxp3 was added, and then incubated in the dark for 40–50 min at 2–8 °C. The cells were washed twice with 2 mL of 1 × Perm/Wash buffer, the supernatant was discarded, and then 350 μL of PBS was added to the tube. Gating for T-cells was performed using CD4^+^ cells. Cell Quest software (BD Bioscience) was used to determine the percentage of CD25^+^/Foxp3^+^ Treg cells in the CD4^+^ cells.

### SKOV3 cell supernatant-exposed lymphocyte responses to mitogen stimulation

Inhibition of T-cell proliferation by SKOV3 cell supernatant was assessed by measuring T-cell proliferation response to stimulation with 10 μg/mL PHA. Briefly, lymphocytes (2 × 10^4^/well) were added to 96-well U bottom plate in the presence of the supernatants of SKOV3 cells treated with or without AO-1 and 1-MT, and RPMI-1640 medium containing 2 % FCS as the control. After 72 h incubation, 20 μL of MTT (5 mg/mL) was added into each well but not into the blank well and incubated at 37 °C, 5 % CO_2_ for 4 h. The plate was centrifuged at 1000*g* for 5 min and the supernatant was removed, and 200 μL of DMSO was added into each well to solubilize the formazan crystals, and then the solution was transferred into 96-well flat bottom plate and absorbance was measured at 595 nm. Proliferation response is calculated as the fomula:

$${\text{Proliferation}}\;{\text{response}}\;\left( \% \right) = {{\left( {Abs_{\text{test}} { - }Abs_{{{{test} /{\text{blank}}}}} } \right)} /{\left({Abs_{\text{control}} { - }Abs_{{{{control} / {\text{blank}}}}} } \right)}} \times 100.$$

### SKOV3 cell supernatant-exposed lymphocyte cytotoxicity

Cytotoxicity mediated by lymphocytes were measured using CytoTox 96^®^ Non-Radioactive Cytotoxicity Assay according to the manufacturer’s protocols. In brief, lymphocytes were preincubated with the supernatants of SKOV3 cells or treated with AO-1 or 1-MT for 24 h and were harvested and resuspended in complete RPMI-1640 medium. Lymphocytes (effector, 1 × 10^4^/well) and Raji or K562 cells (target, cells/well according to effector:target ratios of 1:1, 5:1 and 10:1) were planted into a round-bottom 96-well culture plate in a final volume of 100 μL/well, and mixed gently and centrifuged at 250*g* for 5 min, and then incubated at 37 °C, 5 % CO_2_ for 24 h. Fifty microlitre of the supernatant from each well was transfer to the corresponding well of a flat-bottom 96-well enzymatic assay plate, and then 50 µL of the CytoTox 96^®^ Reagent was added to each well of the plate and incubated at room temperature, protected from light, for 30 min, and 50 µL of the Stop Solution to each well of the plate was added, then absorbance was measured at 490 nm. The percent cytotoxicity for each effector:target cell ratio is calculated as: $\% \;{\text{Cytotoxicity}} = \left[ {{{\left( {{\text{Experimental}} - {\text{Effector Spontaneous}} - {\text{Target Spontaneous}}} \right)} \mathord{\left/ {\vphantom {{\left( {{\text{Experimental}} - {\text{Effector Spontaneous}} - {\text{Target Spontaneous}}} \right)} {\left( {{\text{Target Maximum}} - {\text{Target Spontaneous}}} \right)}}} \right. \kern-0pt} {\left( {{\text{Target Maximum}} - {\text{Target Spontaneous}}} \right)}}} \right] \times 100$

## Results

### AO-1 attenuasstes expression of TLR4/MD-2 complex by SKOV3 cells

**Fig. 1:**
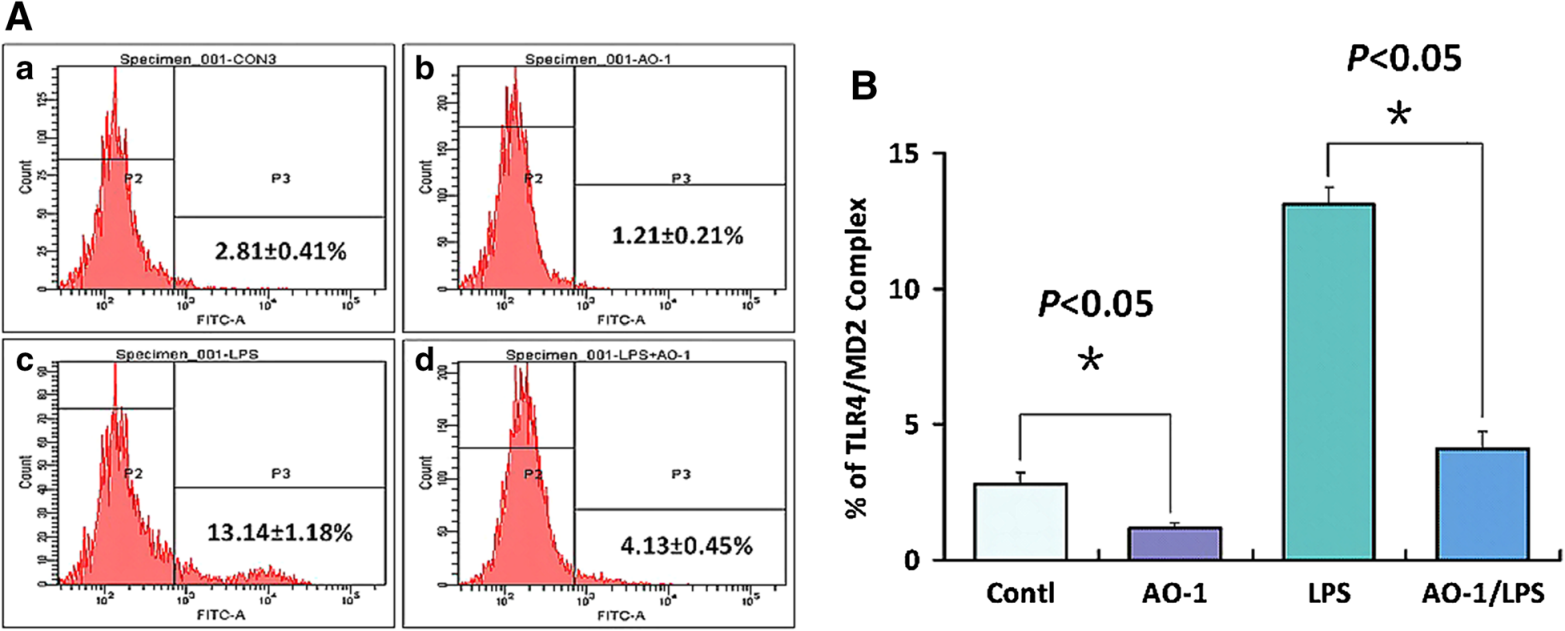
FCM analysis for MD-2/TLR4 complex. **A** Representative FCM diagrams of MD-2/TLR4 complex detection. SKOV3 cells were treated with AO-1(100 μmol/L) alone or combined with LPS (1 μg/mL) respectively. Next, MD-2/TLR4 complex was detected with FCM as described under “Methods” section. **B** Inhibition of AO-1 on LPS-induced MD-2/TLR4 complex in SKOV3 cells. All experiments were performed in triplicate, and data are expressed as mean ± SD (n = 3). *Error bars* represent SD of replicate data points. *P < 0.05, compared to the control

Based on our previous work [33], we propose that binding of AO-1 should inhibit LPS-induced formation of TLR4/MD-2 complex on the surface of SKOV3 EOC cells which express TLR4 and MD-2. FCM analysis showed that SKOV3 cells constitutively express TLR4/MD-2 complex (2.81 ± 0.41 %) (Fig. 1A**-**a), and that the expression of TLR4/MD-2 complex (13.14 ± 1.18 %) was significantly increased following exposure of SKOV3 cells to LPS, compared to the corresponding control (*P* < 0.05) (Fig. 1B); the constitutive and LPS-induced expression of TLR4/MD-2 complex (from 2.81 ± 0.41 to 1.21 % ± 0.21 and from 13.14 ± 1.18 to 4.13 ± 0.45 %, respectively) were significantly decreased following exposure of SKOVE cells to AO-1, as compared to the control and LPS alone (*P* < 0.05) (Fig. 1A**-**b, A**-**c and A**-**d; B).

### AO-1 down-regulates expression and activation of NF-kB and Akt by SKOV3 cells

**Fig. 2:**
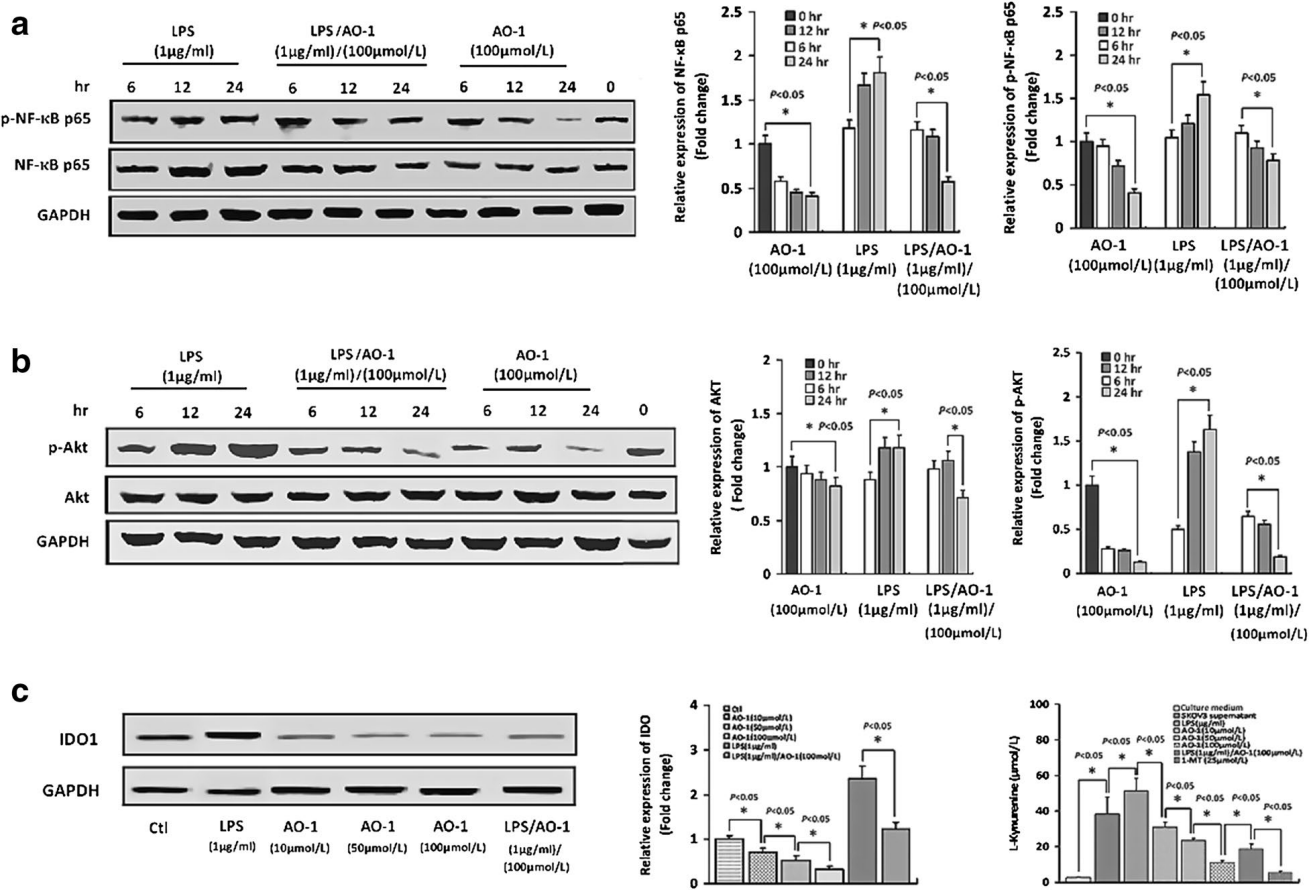
Western blot detection for expression of involved proteins by EOC cells. **a** and **b** SKOV3 cells were treated with AO-1 alone or combined with LPS as indicated concentration for indicated time, and the expression of NF-κB p65, p-NF-κB p65, Akt and pAkt was determined by western blot as described under “Methods” section. **c** Represents IDO1 expression and IDO activity (l-kynurenine release). All experiments were performed in triplicate and data are expressed as mean ± SD (n = 3). *Error bars* represent SD of replicate data points. *P < 0.05, compared to the control

It is important to prove a cross-relationship between activation of TLR4/MD-2 complex and the resultant immunosuppression through constitutively activated NF-κB and Akt signalings in EOC. To address this issue, we examined whether AO-1 could affect the expression and activation of NF-kB p65 and Akt which are required for up-regulation of immunosuppressive factors in MyD88^+^EOC cells. Western blot showed that LPS (1 μg/mL) significantly (*P* < 0.05) increased expression of NF-kB p65, p- NF-kB p65, Akt and p-Akt, and that AO-1 (100 μmol/L) alone or combined with LPS (1 μg/mL) significantly (*P* < 0.05) down-regulated expression of NF-kB p65, p- NF-kB p65, Akt and p-Akt by SKOV3 cells in time-dependent manner (Fig. 2a, b).

### AO-1 down-regulates expression of IDO1 by SKOV3 cells and level of l-kynurenine in SKOV3 cell supernatant

As shown in Fig. 2c, SKOV3 cells constitutively expressed IDO1 protein, and the protein expression of IDO1 was significantly increased (*P* < 0.05) following exposure of SKOV3 cells to LPS but decreased significantly (*P* < 0.05) following exposure of SKOV3 cells to AO-1 alone or combined with LPS, compared to the corresponding control. The IDO1 activity was confirmed by the measurement of l-kynurenine, the breakdown product of tryptophan (Fig. 2c). The level of l-kynurenine in the supernatants was significantly (*P* < 0.05) increased following expoure of SKOV3 cells to LPS but remarkably (*P* < 0.05) decreased following expoure of SKOV3 cells to AO-1 alone or or combined with LPS, compared to the corresponding control. Addition of 1-MT (25 μmol/L) resulted in a significant increase in level of l-kynurenine in the supernatant of SKOV3 cells (*P* < 0.05).

### AO-1 attenuates production of immunosuppressive cytokines in SKOV3 cell supernatant

**Fig. 3:**
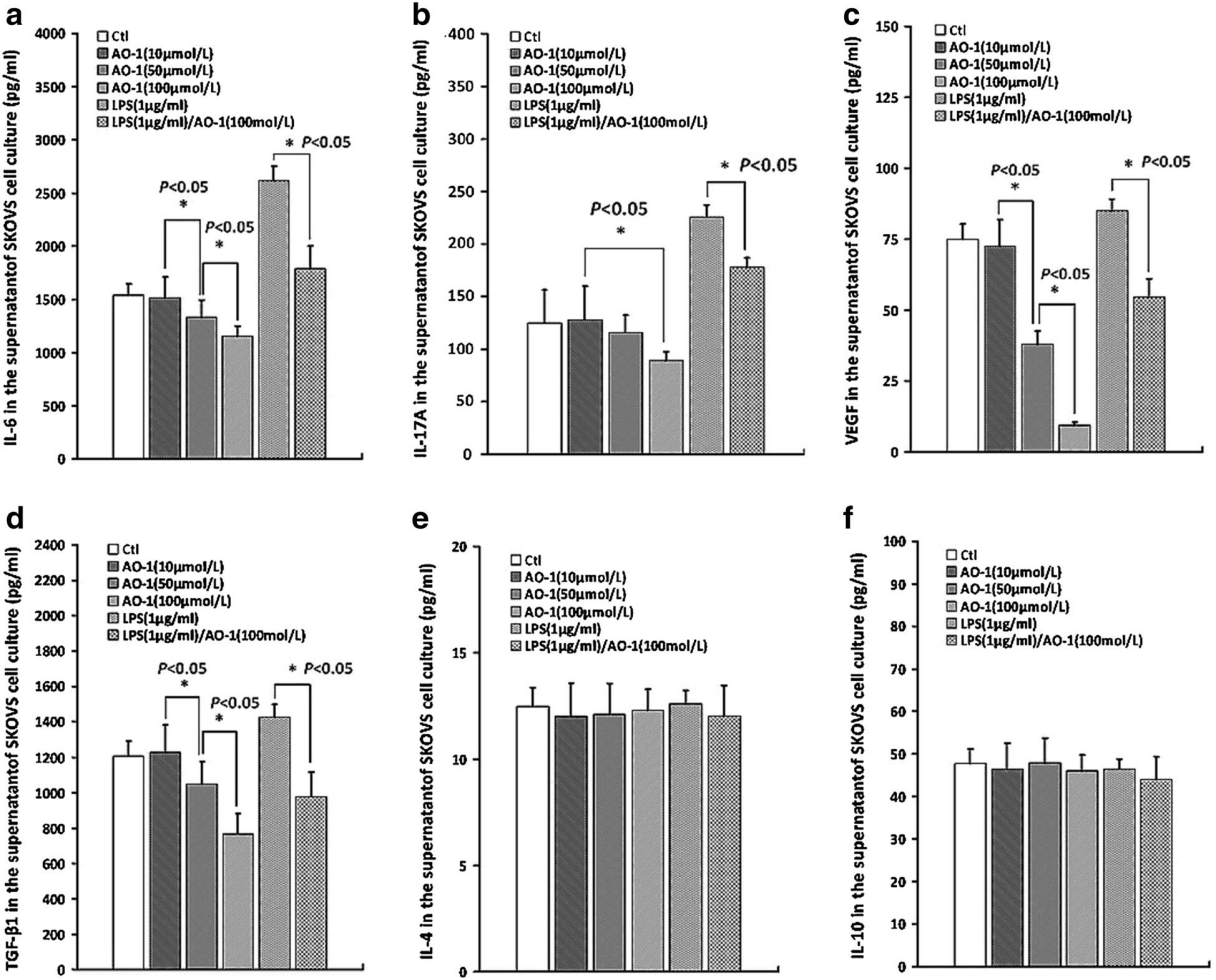
Measurement of cytokines in the culture supernatant of SKOV3 cells. Detection of cytokines in the culture supernatant of SKOV3 cells treated with AO-1 alone or combined with LPS using ELISA as described under “Methods” section. **a**–**f** represents IL-6, IL-17A, VEGF, TGF-β1, IL-4 and IL-10, respectively. All experiments were performed in triplicate, and data are expressed as mean ± SD (n = 3). *Error bars* represent SD of replicate data points. *P < 0.05, compared to the control

To prove whether the down-regulation of NF-kB and Akt activation by AO-1 decreases the production of immunosuppressive cytokines, the supernatants of SKOV3 cells exposed to LPS with or without addition of AO-1 for 48 h were analyzed for IL-6, IL-17A, IL-10, IL-4, TGF-β1 and VEGF by ELISA. As shown in Fig. 3, SKOV3 cells constitutively secreted IL-6 (1539 ± 107.73 pg/mL), IL-17A (116 ± 29 pg/mL), IL-10 (47.8 ± 3.35 pg/mL), IL-4 (12.6 ± 0.63 pg/mL), TGF-β1 (1205 ± 84.35 pg/mL) and VEGF (75 ± 5.25 pg/mL). In response to LPS (1.0 μg/mL), the levels of IL-6 (2618 ± 130.9 pg/mL), IL-17A (226 ± 11.3 pg/mL), TGF-β (1425 ± 73.25 pg/mL) and VEGF (85 ± 4.08 pg/mL) were remarkably increased, compared to the control (*P* < 0.05); Whereas AO-1 not only decreased the constitutive but also LPS-induced levels of IL-6, IL-17A, TGF-β1 and VEGF (*P* < 0.05) (Fig. 3a–d), but did not impact the levels of IL-10 and IL-4 (Fig. 3e, f), as compared to the corresponding control.

### AO-1 modulates the subpopulations of T lymphocytes exposed to SKOV3 cell supernatant

**Fig. 4:**
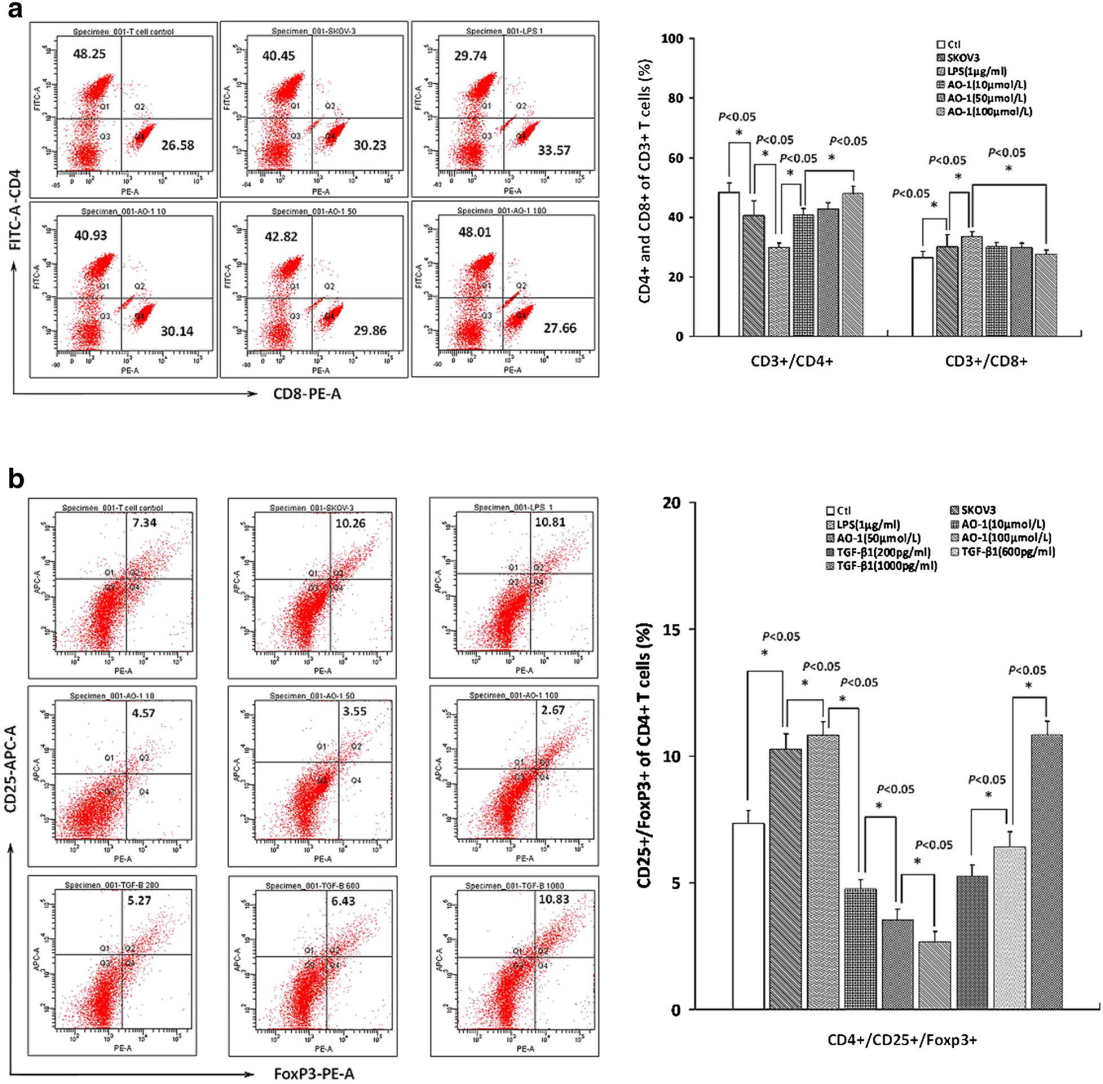
Detection of subpopulation of T cells and Treg cells of lymphocytes. Detection of subpopulation of T cells and Treg cells of lymphocytes exposed to the culture supernatant of SKOV3 cells treated with AO-1 alone or combined with LPS using FCM assay as described under “Methods” section. **a** FCM diagrams of CD3^+^/CD4^+^/CD8^+^ of T cells, and **b** FCM diagrams of CD4^+^CD25^+^FoxP3^+^ of Treg cells. All experiments were performed in triplicate, and data are expressed as mean ± SD (n = 3). *Error bars* represent SD of replicate data points. *P < 0.05, compared to the control

As shown in Fig. 4a, CD4^+^/CD8^+^ ratios of T cells were 1.34 ± 0.17 and 0.86 ± 0.12, respectively, and higher than that (1.81 ± 0.24) in the control following exposure of lymphocytes to the supernatants of SKOV3 cells or treated with LPS, but were 1.36 ± 0.18, 1.43 ± 0.19 and 1.74 ± 0.23, respectively following exposure of lymphocytes to the supernatants of SKOV3 cells treated with AO-1 (10, 50 and 100 μmol/L); As shown in Fig. 4b, CD4^+^CD25^+^Foxp3^+^ levels of T cells were 10.26 ± 0.62, 10.81 ± 0.65 and 10.83 ± 0.72 %, respectively, and were higher than that (7.34 ± 0.51 %) in the control following exposure of lymphocytes to the supernatants of SKOV3 cells or treated with LPS and 1000 pg/mL of TGF-β1 as a positive control, but were 4.75 ± 0.33, 3.55 ± 0.24 and 2.67 ± 0.24 %, respectively following exposure of lymphocytes to the supernatants of SKOV3 cells treated with AO-1 (10, 50 and 100 μmol/L). AO-1 significantly improved the CD4^+^/CD8^+^ ratio but decreased the CD4^+^/CD25^+^/Foxp3^+^ levels of T cells in a concentration-dependent manner, as compared to the corresponding control (*P* < 0.05).

### AO-1 improves proliferative response of T lymphocytes exposed to SKOV3 cell supernatant

**Fig. 5:**
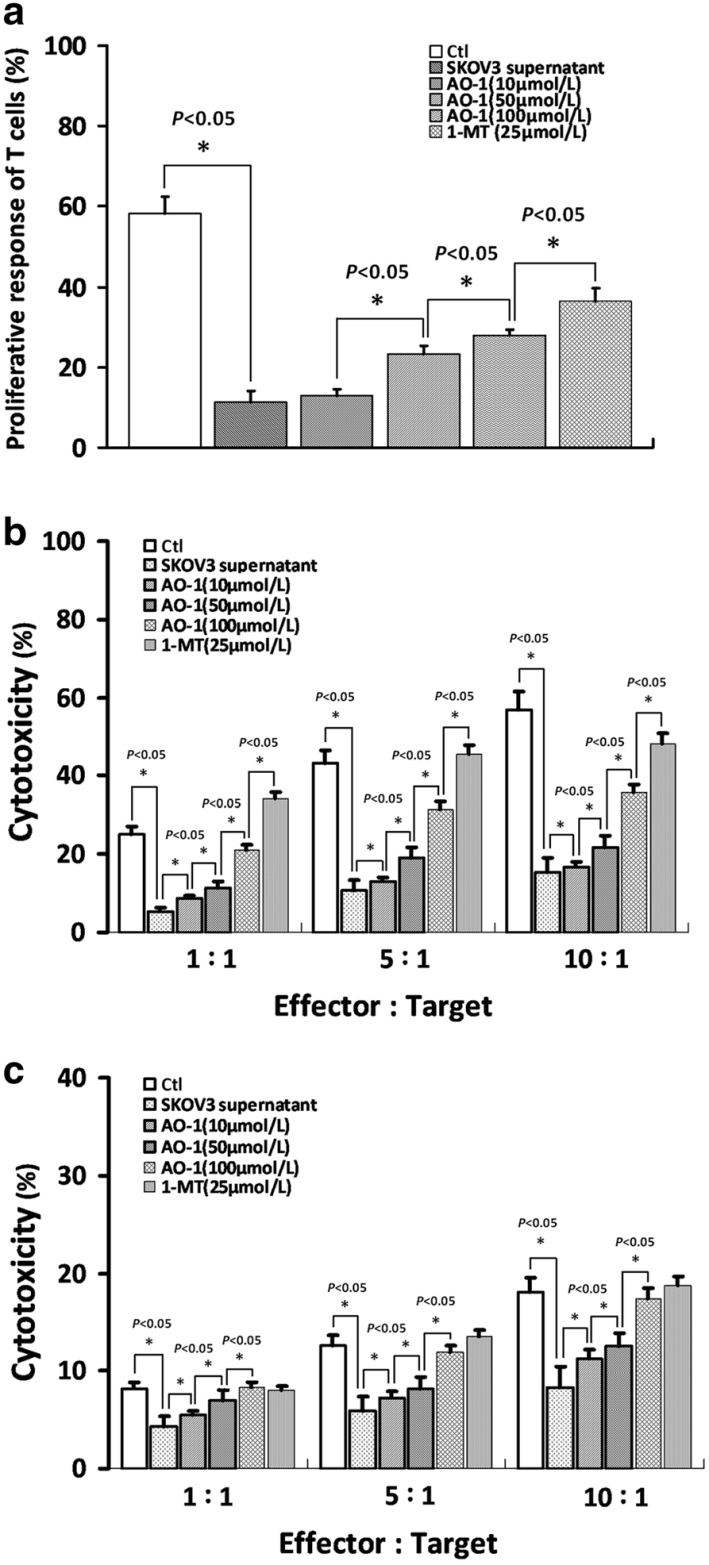
Detection of proliferative response and cytotoxicity of T cells. **a** Represents the detection of proliferative response of T cells with PHA stimulation using MTT assay as described under “Methods” section, and **b**, **c** the detection of T cells cytotoxicity against K562 and Raji cells following exposure of lymphocytes to the supernatant of SKOV3 cells treated with AO-1 or 1-MT using LDH release assay as described under “Methods” section, respectively. All experiments were performed in triplicate, and data are expressed as mean ± SD (n = 3). *Error bars* represent SD of replicate data points. *P < 0.05, compared to the control

As shown in Fig. 5a, the proliferative responses of T lymphocytes were reduced following the supernatant of SKOV3 cells but significantly improved following exposure of lymphocytes to the supernatant of SKOV3 cells treated with or 1-MT, compared to the corresponding control (*P* < 0.05). The T lymphocytes exposed to supernatant of SKOV3 cells had significantly lower responses to PHA and lost the ability to respond to mitogens in the PHA stimulation system.

### AO-1 improves antitumor cytotoxicity of T lymphocytes exposed to SKOV3 cell supernatant

As shown in Fig. 5b, c, the killing effects of T lymphocytes on K562 and Raji cells were significantly impaired following exposure of lymphocytes to the supernatant of SKOV3 cells but significantly ameliorated following exposure of lymphocytes to the supernatant of SKOV3 cells treated with AO-1 or 1-MT, compared to the corresponding control (*P* < 0.05).

## Discussions

A major obstacle for the development of effective immunotherapy is the ability of tumors to escape the immune system. Cancer is the initiator of evil in the immunosuppressive microenvironment. To be effective, EOC immunotherapy may be necessary to reversion of cancer cell-mediated immunosuppression. Presently EOC immunotherapy remains behind. Characterization of EOC cells that express TLR4/MD-2 and MyD88 have helped to confirm the dynamic role of the immunosuppressive microenvironment in promoting cancer metastasis and recurrence. One of the major reasons for the insufficient response to current immunotherapies is thought to be tumor-mediated immunosuppression, which is triggered by oncogene activation and dysregulated signaling in cancer cells. However, the mechanisms leading to the production and induction of immunosuppressive molecules are not fully understood. Some anti-inflammatory phytochemicals appear to exhibit activity in modulating the tumor microenvironment. Phyto-active compounds have been shown to induce apoptosis, and prevent or delay chemotherapy-resistance. Specifically curcumin and quercetin as a ligand of MD-2 or TLR4 potently repress TLR4/MyD88/NF-кB signal pathway and inhibit immunosuppressive cytokine production, and suppress ovarian cancer cell metastasis. The experiments described herein aimed to clarify the relationship between the immunosuppression and TLR/MD-2 complex-mediated activation of MyD88/NF-κB signaling in human EOC, as well as to develop an immune modulatory therapy targeting TLR/MD-2 complex. Human EOC cells usurp or hijack TLR4/MyD88 signaling pathway that considerably activates NF-κB and Akt pathways, which trigger expression of immunosuppressive molecules resulting in immune incompetence in the tumor microenvironment [6, 13]. TLR4/MD-2 complex plays a crucial role for activation of MyD88/NF-κB pathway in human EOC cells. In this study, we have shown that AO-1 could depress LPS-induced increased expression of TLR4/MD-2 complex by blocking assembly of TLR4/MD-2 (Fig. 1A, B), and down-regulate LPS-induced IDO1 in EOC SKOV3 cells (Fig. 2c). LPS is an agonist of TLR4/MD-2 complex, and MD-2 is requisite for the activation of TLR4/MD-2 complex and directly bridges the two components of the multimer resulting in TLR4 coupling MyD88 [14–16]. AO-1 is an antagonist of TLR4/MD-2 complex, just like Eritoran [34, 35], and may block MD-2-bridged TLR4 homodimer coupling with MyD88, resulting in inhibition of MyD88-dependent signaling pathway that activates NF-κB signaling. A functional TLR-4/MyD88/NF-κB pathway confers to EOC cell the capacity to respond to TLR4 ligands and enhances NF-κB activity and cytokine production, and plays a central role in the control of immunosuppression in human EOC [16]. We found that AO-1 down-regulated LPS-induced the phosphorylation of NF-κB p65 and Akt (Fig. 2a, b), suggesting that the activation of NF-κB p65 and Akt is dependent on TLR4/MD-2 complex in SKOV3 EOC cells. EOC cells are able to induce expression of FoxP3 in CD4^+^/CD25^+^ T regulatory cells and exhibit suppressive ability in activated naïve T cells by producing soluble multiple cytokines. Constitutive expression of IL-6, TGF-β1, IL-17A, VEGF, IL-4, IL-10 and IDO by tumor cells as a major component of immune escape and immunosuppression in human EOC. It has been demonstrated that the ascites IL-6 of EOC patients influences the local immunity and contributes to lower fraction of NK cells and CD8^+^/CD3^+^ cells [25]; Both of TGF-β1 and IL-17 induce an increase in frequencies of Treg cells (CD4^+^CD25^+^FoxP3^+^) and the number of FoxP3^+^ cells is positively correlated with the immunoexpression of IL-17 and TGF-β1. Similar to TGF-β1, the supernatant derived from SKOV3 cells could convert part of freshly isolated CD4^+^/CD25^−^ T cells into CD25^+^ population with characters as CD4^+^/CD25^+^/FoxP3^+^ Treg cells. Knockdown TGF-β1 gene increases the immunogenicity of human EOC cells and impairs the tumorigenic ability of human EOC cells [36, 37]. The role of VEGF in tumor angiogenesis has been well characterized, nevertheless, it is also known to have an immunosuppressive activity besides its angiogenic role and to promote tumor immune escape by impairing DC maturation and antitumour T cell activation in the tumor microenvironment. Recent studies show that VEGF significantly reduces the cytotoxic activity of T cells and directly suppresses T cell activation via VEGF receptor type 2, and also inhibits LPS-induced maturation of DCs [38, 39]. In patients with EOC, EOC cells are able to synthesize and secrete IL-10 and IL-4. It has been documented that proinflammatory stimuli IL-1β and TNF-α enhance IL-10 secretion, but LPS and IL-6 have no influence on the release of IL-10 [19], whereas IL-10 and IL-4 do not influence elevated FoxP3 expression induced by EOC cell culture supernatant [40]. These results demonstrate that AO-1 could down-regulate expression of IL-6, TGF-β1, IL-17A and VEGF (Fig. 3a–d) but not IL-4 and IL-10 by SKOV3 cells (Fig. 3e, f), and reduce frequencies of Treg cells following exposure of lymphocytes to the supernatant of SKOV3 cells alone or treated with LPS, suggesting that the expression of IL-6, TGF-β1, IL-17A and VEGF is dependent on TLR4/MD-2/MyD88/NF-κB and Akt signaling pathways. miR-155 has been shown to influence CD4 T cell, regulatory T cell and effector and effector memory CD8 T cell differentiation [40]. The Foxp3 target miR-155 contributes to the development of regulatory T cells. miR-155 expression is strongly induced by inflammatory cytokines [41, 42]. Human epithelial ovarian carcinoma cell-derived cytokines may cooperatively affect on miR-155 expression of T-cells, contributing to changes of T cell phenotype and function [43].

It is known that IDO plays a critical regulatory role in EOC progression [28, 44]. IDO, as an inducer and amplifier of Treg cell functions, activates regulatory T cells. Evidence emerges indicating that IDO possibly promotes tumor immune escape by inducing an immunoregulatory T cell phenotype at a systemic level, and allogeneic T cell proliferation is inhibited by IDO-expressing cancer cells [45]. IDO can both deplete tryptophan in local tissue microenvironments and generate kynurenine, a tryptophan catabolite. Kynurenine is known to induce the conversion of naïve CD4^+^/CD25^−^ T cells into highly suppressive CD4^+^/CD25^+^/Foxp3^+^ Treg cells and to inhibit the proliferative response of T cells to mitogen stimulation. Treg cell is known to induce IDO expression in DCs and to change from inflammatory to regulatory DCs, which can in turn enlarge the Treg cells compartment by tryptophan catabolism [46]. IDO promotes the peritoneal dissemination of EOC by inhibiting NK cell accumulation in tumors and promoting angiogenesis. IDO gene silencing in EOC SKOV3 cells transfected with siRNA reduced the constitutively expresses IDO and release of kynurenine into the supernatant, and suppressed tumor progression and peritoneal dissemination and enhanced the sensitivity of cancer cells to NK cells in the tumor microenvironments. Down-regulation of IDO controls human EOC progression by activating NK cells [28]. The constitutive IDO expression in human EOC is sustained by an IL-6 autocrine signaling loop that is critical for IDO-mediated immunosuppression in human EOC [29]. These data represented more common phenomenon of immunosuppression of tumor microenvironment in human EOC. The tryptophan metabolite kynurenine inhibits cell growth and induce apoptosis. T cells are particularly sensitive to this stress, which easily suppresses their function [46]. In IDO-expressing tumors, IDO promotes local tryptophan degradation and depletion, resulting in T-cell function suppression, leading to local immunotolerance. In the present study, we showed that AO-1 downregulated IDO protein expression (Fig. 2c) and functional activity by EOC cells and increased the proliferative response of T cells to mitogen stimulation (Fig. 5a) and the sensitivity of cancer cells to T cells and NK cells in vitro (Fig. 5b, c). The possibility cannot be excluded that TLR4/MD-2 complex expression is involved in EOC immunosuppression through such an IDO-mediated mechanism. These findings indicate that the AO-1 targeting TLR4/MD-2 complex modulates IDO-mediated immunosuppression in SKOV3 cells.

## Conclusion

We have demonstrated that TLR4/MD-2 complex-mediated activation of MyD88/NF-kB is the major mechanism for immunosuppression through induction of immunosuppressive molecules and generation of immunosuppressive T cells by human EOC SKOV3 cells. TLR4/MD-2 complex inhibitor such as AO-1 may be useful for reversal of immunosuppressive conditions in EOC, which may increase responses to immunotherapy and chemotherapy. This study suggested that TLR4/MD-2 complex could potentially serve as a target in reversion of the immunosuppressive microenvironment of human EOC and the combination immunotherapy targeting TLR4/MD-2 complex may be an attractive strategy for MyD88-expressing EOC treatment. Certainly, the role of TLR4/MD-2 complex for activation of MyD88/NF-κB signaling needs to be further studied in a variety of ovarian cancer cell lines of different phenotypes, and even in vivo study.
